# COVID-19 and Orthopaedic International Humanitarianism

**DOI:** 10.5435/JAAOSGlobal-D-20-00222

**Published:** 2021-02-10

**Authors:** Alec J. Talsania, Chris Lavy, Harpal S. Khanuja, Hank Chambers, Nancy A. Kelly, Richard O. E. Gardner, Scott Nelson, Biruk L. Wambisho, Francel Alexis, Donald H. Lalonde, R. Richard Coughlin, J. Turner Vosseller, Eric C. Gokcen

**Affiliations:** From Lewis Katz School of Medicine at Temple University, Philadelphia, PA (Mr. Talsania); the Nuffield Department of Orthopaedics, Rheumatology and Musculoskeletal Sciences, University of Oxford, Oxford, UK (Professor Lavy); the Department of Orthopaedic Surgery, Johns Hopkins University, Baltimore, MD (Dr. Khanuja); Rady Children's Hospital, San Diego, CA (Dr. Chambers), and Department of Clinical Orthopaedics, University of California at San Diego, San Diego, CA (Dr. Chambers); Health Volunteers Overseas, Washington, DC (Ms. Kelly); CURE International and CURE Ethiopia Children's Hospital, Addis Ababa, Ethiopia (Dr. Gardner); the Department of Orthopaedic Surgery, Loma Linda University School of Medicine, Loma Linda, CA (Dr. Nelson), and Haiti Adventist Hospital, Carrefour, Haiti (Dr. Nelson); the Department of Orthopaedics, Addis Ababa University, Addis Ababa, Ethiopia (Dr. Wambisho), and Black Lion Hospital, Addis Ababa, Ethiopia (Dr. Wambisho); the Department of Orthopedics, Haiti Adventist Hospital, Carrefour, Haiti (Dr. Alexis); Dalhousie University, New Brunswick, Canada (Dr. Lalonde); the Department of Orthopaedic Surgery, University of California San Francisco, San Francisco, CA (Dr. Coughlin); the Department Foot and Ankle Surgery, Jacksonville Orthopaedic Institute, Jacksonville, FL (Dr. Vosseller); and the Department of Orthopaedic Surgery, Department Foot and Ankle Surgery, and the Department of Orthopaedic Surgery and Sports Medicine, Lewis Katz School of Medicine at Temple University, Philadelphia, PA (Dr. Gokcen).

## Abstract

As the world continues to adjust to life with COVID-19, one topic that requires further thought and discussion is whether elective international medical volunteerism can continue, and, if so, what challenges will need to be addressed. During a pandemic, the medical community is attentive to controlling the disease outbreak, and most of the literature regarding physician involvement during a pandemic focuses primarily on physicians traveling to areas of need to help treat the disease. As a result, little has been written about medical volunteerism that focuses on medical treatment unrelated to the disease outbreak. In a world-wide pandemic, many factors are to be considered in determining whether, and when, a physician should travel to another region to provide care and training for medical issues not directly related to the pandemic.

Leaders of humanitarian committees of orthopaedic surgery subspecialties engaged with one another and host orthopaedic surgeons and a sponsoring organization to provide thoughtful insight and expert opinion on the challenges faced and possible pathways to provide continued orthopaedic support around the globe. Although this discussion focuses on international orthopaedic care, these suggestions may have a much broader application to the international medical community as a whole.

In 2016, an estimated 200,000 American global health volunteers traveled to various places around the world to provide medical assistance, with an estimated annual expenditure of $250 million.^1,2^ Although it is arguable whether all of these volunteers were necessary or whether they provided any measurable improvement in healthcare outcomes, most would agree that some level of international medical volunteerism is indeed helpful for the medical care in low- and middle-income countries (LMICs).^3,4^ In addition, most aid travelers feel that it is their moral duty to help those in need, regardless of where they may be on the globe.^5^

The novel coronavirus (COVID-19) has impacted the daily lives of people around the world, in both LMICs and high-income countries (HICs) as cases have surpassed 75 million across 191 countries.^6^ The effects in LMICs have been particularly profound because COVID-19 has disrupted immunizations services, worsened human trafficking situations, and contributed to social unrest.^7-9^ The pandemic has also placed a burden on medical systems in LMICs, where healthcare facilities were already limited in resources and overwhelmed with patients suffering from transmissible diseases, such as malaria, tuberculosis, and pneumonia.^10-12^ In addition, LMICs are less capable of delaying surgeries to account for increased hospitalizations, as was done in HICs, because the surgical volume is largely urgent and cannot be easily postponed.^10^ LMICs are in even greater need because of the shifting of resources to manage the pandemic, and thus, nonpandemic care may get postponed.

Despite the anticipated rapidly increasing need for global health volunteerism because of the COVID-19 pandemic, many questions arise when considering the resumption of international medical service trips. The purpose of this study was to obtain expert opinion from some of the leaders of orthopaedic international humanitarianism to discuss these challenges and suggest pathways to provide continued orthopaedic support around the world.

## Why We Go

The COVID-19 pandemic has closed down all but essential travel, and many medical volunteers have had to cancel or postpone service trips. Although frustrating, setbacks always bring opportunities for reflection. It is therefore first worth asking why so many medical volunteers pursue humanitarian trips.

The enormous clinical need motivates many to volunteer. A tremendous shortage exists in both manpower and expertise available to treat essential and emergency conditions in LMICs, with evidence that the deficits may be worsening.^13-15^ Surgeons from HICs can have a great impact in LMICs by providing surgical care that would otherwise be limited or unavailable. Volunteers can also play a role in assisting local trainers or freeing them up for other tasks. Providing services that are in addition to the work done by the host surgeons can at least temporarily increase surgical capacity.

A similar need exists for the training of healthcare personnel. Education allows the national healthcare system to improve its standards and can lead to better healthcare outcomes for the population through the development of competent surgeons. Indeed, without this increase in capacity moving forward, the population will not have adequate access to the care they need. Thus, it is paramount that volunteers work with local surgeons to improve and advance the level of teaching.

From an individual perspective, some volunteers appreciate learning about new conditions that are only seen in LMICs. For example, untreated clubfoot is far more common in LMICs, and the volunteer can observe the natural history of the condition in addition to its management in an advanced state. Other volunteers provide care based on their faith, which encourages them to care for those less fortunate. Many nongovernment organizations (NGOs) in LMICs are faith based, working to provide humanitarian healthcare in areas with great need.

## Volunteer Perspective

With COVID-19 appearing in countries all around the world, new factors must be considered when making a decision to volunteer abroad. As with any volunteer assignment, preparation is key. Volunteers must work with the hosts to assess the site's situation, needs, the local impact of COVID-19, and whether the presence of a volunteer will be an asset or a liability.

Each prospective volunteer must first take stock of his or her own situation by assessing their health and whether it is an appropriate time to travel. It is better to err on the side of caution if the volunteer has any underlying health conditions. In addition, dealing with the risks inherent to traveling must be factored into any decision to volunteer abroad. Whether it is terrorism, HIV/AIDs, Ebola, Zika, avian flu, or in this case COVID-19, volunteers have to weigh the risks, take precautions, and work within established safety guidelines. Situations can change quickly, and volunteers need to be flexible. They should closely follow local news updates while staying in constant communication with the project site and their sending organization.

Prospective volunteers should also consider their work situation at home. It is possible that volunteering abroad during the pandemic will create more of a hardship for their colleagues and patients. Furthermore, the pandemic and its economic fallout may have caused financial or personnel constraints that would be exacerbated if a volunteer spends time away. The temporary shutdown of services during the pandemic has created a backlog of patient demand for urgent services at home that still need to be addressed.

Visa policies are changing, and countries are barring certain nationalities from entering if they are traveling from countries where the COVID-19 caseload is high (such as the United States). Countries allowing visitors often have a 2-week quarantine policy in effect. This severely limits time for volunteering and sharing skills that instead may be spent in government custody or the confines of a hotel room. In addition, some states in the United States are requiring returning travelers to quarantine for 2 weeks at home. The time spent in quarantine, both at the project site and on returning, may have financial consequences for volunteers who are not able to work during that time.

Volunteers should consider purchasing travel insurance and monitor airline requirements frequently because some airlines are requiring a negative COVID-19 test. Some countries require a substantial financial deposit to ensure that any emergency medical procedures for COVID-19 would be covered. In addition, many countries are not allowing travelers to enter, and others are allowing limited transit times of only a few hours.

If a volunteer is able to travel to a project site, the availability of personal protective equipment (PPE) may be very limited or of questionable quality. Volunteers may choose to bring their own PPE but that poses an ethical dilemma if their colleagues at the project site are not as well equipped. Should volunteers bring their own equipment, they should consider bringing enough for their hosts as well, and plan to leave any reusable PPE when they depart.

Volunteers should recognize that COVID-19 testing in the host country may be limited or unavailable. If testing is in short supply, priority should go to the host country providers. Volunteers may choose to get tested before leaving for their assignment, but traveling for long hours may result in exposure, which may be asymptomatic for the initial days at the project site. If a volunteer becomes ill with COVID-19, this could lead to serious ramifications. The volunteer may be viewed as having “imported” the disease to the country, and there may be backlash against the volunteer, the sponsoring organization, and the country where both are located.

Recognizing all of these concerns and risks, some volunteer organizations, such as Health Volunteers Overseas, have shifted to an alternative service model for the duration of the pandemic. Rather than having volunteers serve at project sites, they are providing virtual education and training through live and recorded lectures and demonstrations, reviews of articles for publication, and mentoring of colleagues abroad. These efforts have proven to be both effective and meaningful, as well as maintaining collegial support to colleagues during this difficult time.

## Host Perspective

In the past, we have seen the considerable transformative changes that visiting teams can bring to the surgical care in host countries. In this current uncertain time, we advocate for a careful assessment of the goals of the individual mission trip but do not wish to see the pendulum swing too far toward a sustained postponement of valuable support for surgical programs in LMICs.

COVID-19 has initially affected many LMICs less than what was expected. Social overcrowding, oversubscribed local transport, and limited access to running water were expected to result in a rapid and severe escalation in case numbers that would overwhelm local healthcare resources. Africa seems to have been spared the brunt of the pandemic, which can only partially be explained by limited access to testing.^16^ Additional theories to explain this include a younger population, less obesity, heart disease and diabetes, and possibly a more robust immune system because of a wide exposure to microorganisms from a young age. Natural ventilation and spending large amounts of time outdoors may also be factors.

Any early resentment in the pandemic toward visitors seems to have resolved in many countries. This may be due to established local community transmission, stringent precautions for those arriving from abroad, and/or a perception of decreased susceptibility in populations that have largely been spared from the pandemic. Despite this, local governments are still taking careful measures to minimize the risk posed by visitors. Some countries maintain closed borders, whereas others have a mandatory 2-week quarantine for all arrivals. Some nations allow avoidance of quarantine for travelers who have a negative polymerase chain reaction result from a few days before travel. The risk to the host institution should be minimized by following the national guidelines for arrivals and standard precautions regarding testing, mask wearing, social distancing, and using proper hand hygiene.

In the event of further escalation of critical pandemic cases, the surge capacity of local intensive care and high dependency units is limited. In Haiti, for example, minimal surge capacity exists, and treatment for COVID-19 in this environment is supportive only. In the seven countries where CURE International has hospitals (central and southern Africa and the Philippines), the steady escalation to date has not overwhelmed the government-run high dependency healthcare resources. Most healthcare institutions in Africa reacted in a similar way to hospitals in Europe and North America with widespread cancellation of elective surgical services, creating a financial hardship for these facilities. Some tertiary referral centers in capital cities, which are typically COVID-19 hotspots, have also needed to make sensible decisions to limit patients traveling from rural areas who could be a vector for COVID-19 spread on returning home.

Thus, for the volunteer, the concept of “first do no harm” still applies during the dynamics of this pandemic. Because Africa has seen a late escalation in COVID-19 cases compared with the United States and Europe, we need to weigh the risks and benefits of travel not just for the visitor but also for the hosts. Currently, that would mean only the most essential trips should proceed. However, once the rate of new cases adequately declines and further waves are not anticipated, routine travel should again be possible. There may be some leveling of the prevalence of active infection rates between HICs and LMICs, and any remaining differences may become better understood with antibody tests and further research. Global interaction will hopefully be increased further as vaccines become widely available.

As the initial wave passed, demand for surgical care returned close to the prepandemic volume. Although the reality of a second wave has come and the possibility of future spikes lingers, it remains true that volunteer surgeons may have a skill set that is unique for the host country. Patients will often be placed on a waiting list for a visiting specialist surgeon. It is our experience that patients will often prefer to wait and take advantage of this opportunity to access the specialized care that they need.

In relation to partner-host orthopaedic collaborations seen during the pandemic, a shift has been noted to sending PPE equipment in addition to orthopaedic supplies and training materials. Partners have developed online teaching tools using technology not previously used in LMICs. Quarantine restrictions of volunteers have limited their face-to-face interaction, but consultations can be done via local phone calls. Many scholarships, visits, and short-term trainings have been postponed; however, through frequent communications, replanning efforts can be explored.

Those of us who live and work in LMICs would not want to see vital support of programs compromise patient care in the medium to long terms because of COVID-19. International expertise is valuable for training, patient care, service development, and capacity building. A balance certainly exists, and wisdom is needed, but a risk of losing valuable ground exists if international support pulls back too far.

## Opportunities for Change

An accepted definition of “opportunity” is a “set of circumstances that make it possible to do something.”^17^ COVID-19, given its profound global effect, inequitable impact, and barriers to travel, provides this moment for us to truly do something. Historically, scholars and practitioners of global outreach and humanitarianism have articulated key principles to consider and follow as we develop our helping strategies. As surgeons, we understand “first do no harm,” but further, the goals of empowerment and sustainability direct us away from mission trips toward education, training, and systems building. More recently, in consideration of the striking importance of awareness of equity and decolonization, this opportunity allows us to reimagine our outreach and humanitarian work through the lens of equity, bilateral reciprocity, and mutual respect.

Historically, most outreach opportunities have traveled to provide surgical service with or without an educational component, which has been previously challenged.^18^ With COVID-19 restrictions, a myriad of ways exist to be involved and make a difference without the need for travel or scholarly exchange. Perhaps, the best place to start is by simply asking your established partners “How can I best help?” Solidarity and friendship can be indispensable, especially when shared challenges exist. Frequently, their needs may be quite different and less costly than what we might imagine.

For those of us who may not have local contacts, established NGOs or organizations with a long history and experience with overseas work can provide some guidance. Health Volunteers Overseas has been a long-standing successful volunteer NGO with a focus on the teaching/training model of intervention. Furthermore, they have the resources and relationships that may point you in the right direction.^19^

Increasingly, many US academic programs have established relationships and programs and may welcome your involvement through volunteer or philanthropic support. The Institute for Global Orthopaedics and Traumatology (IGOT), an academic initiative within the Department of Orthopaedics at the University of California, San Francisco, has focused on an “academic to academic model.”^20^ IGOT provides an online educational portal, supports locally relevant research, and promotes leadership development. IGOT has adapted its in-country Surgical Management and Reconstruction Training course to be provided online. A further expansion of this concept has been the organization Consortium of Academic Traumatologists, in which 30 member institutions promote global musculoskeletal trauma care through sharing best practices, research, mentorship, and resources.^21^ SIGN Fracture Care International is another well-established NGO that has built sustainable orthopaedic capacity by providing education to surgeons while also manufacturing and donating instruments and implants.

Most professional orthopaedic societies have now embraced global outreach into the fabric of their organizations. As an example, the Orthopaedic Trauma Association has developed an extensive online educational portal and has provided subscriptions to access this knowledge through supporting a number of LMICs academic teaching programs.^22^ Another activity that can be done from home is further production of educational content along with curriculum building through each musculoskeletal subspecialty.

Technology has created an enormous potential for innovation, along with cost-effective and sustainable solutions to education, training, and professional development. One generic online educational modality has been the proliferation of webinars and online courses. The preponderance of this effort has been directed at more state-of-the-art educational material and techniques targeted to developed-world surgeons. Although certain topics are generalizable and pertinent to basic principles and evidence base, there exists a need for more targeted educational material for LMIC surgeons.

American Society for Surgery of the Hand has successfully developed such targeted education through webinars.^23^ After a questionnaire was sent to 338 surgeons from 27 LMICs asking their preference of content, a Webinar was presented on tendon transfers. Another excellent example of an appropriate technology was a Webinar from the outreach organization of the British Society for Surgery of the Hand advocating for the use of WALANT (wide awake local anesthesia no tourniquet) for limb surgery. This technique is especially effective in less resourced LMIC operating rooms because it decreases the surgical risk of spreading COVID-19 from intubation.^24^

The advent and necessity for telemedicine within our own COVID-19 constraints can only be amplified by the provision of consultations and sharing of challenging cases with our global colleagues. This bidirectional professional exchange benefits the musculoskeletal care of not only patients abroad but also at home. E-mail sharing has now been augmented with the use of more effective video-conferencing such as Skype, Zoom, GoTo Meeting, and Cisco WebEx.

COVID-19 has provided an opportunity to shift global outreach from “giving a man a fish” to “teaching a man to fish” through digital education and appropriately targeted use of technology, enabling our partners to drive their own change through empowerment. These outreach interventions may have lasting impacts at many levels—individual, community, programmatic, societal, institutional, health system, and ministry of health.^25^ The opportunity to “do something” has never been better!

## Conclusion

It is fair to say that none of us could have predicted that we would be collectively fighting against a disease that has affected virtually every nation in the world. Our best defense remains the bulwark of our foundation, relying on science and data to increase our understanding and to direct our next steps. As volunteers, we start by clearly defining why we volunteer in order to reassess our role in light of the current pandemic. It is equally important to understand volunteerism from the host perspective, and how COVID-19 has changed the facts on the ground in LMICs. With a clear understanding of these factors, we must then consider the practicality and safety of volunteering abroad during a pandemic. Finally, we must call on our collective human capacity for adaptation and recognize and seize those opportunities that the pandemic may have afforded us. Flexibility and creativity can flourish in this space, although, as with the goal of teaching a man to fish, the goal of a volunteer in this setting is ultimately to make himself or herself obsolete.
